# “Unique trend” and “contradictory trend” in discrimination of primary synchronous lung cancer and metastatic lung cancer

**DOI:** 10.1186/1471-2407-13-467

**Published:** 2013-10-09

**Authors:** Cheng Shen, Huan Xu, Lunxu Liu, Yubin Zhou, Dali Chen, Heng Du, Zhaojie Han, Guowei Che

**Affiliations:** 1 Department of Cardiovascular and Thoracic Surgery, West-China Hospital, Sichuan University, Chengdu 610041, China; 2 Department of Pathology, West-China Hospital, Sichuan University, Chengdu 610041, China

**Keywords:** Primary synchronous lung cancer, Metastatic lung cancer, Microsatellite analysis

## Abstract

**Background:**

Distinguishing between multiple primary lung cancers and metastatic tumors is often difficult when the tumor histology is same. Since genomic instability is a common feature of cancer, we hypothesized that independently arising neoplasms in an individual patient would exhibit measurable genomic variation, enabling discrimination of tumor lineage and relatedness. The feasibility of analyzing genomic instability expression profiles to distinguish multiple primary lung cancers from metastatic tumors was evaluated.

**Methods:**

This study enrolled 13 patients, with multiple primary lung cancers demonstrating with the histology, who underwent surgery between April 2003 and December 2012 at the Department of the Thoracic Surgery at West China Hospital in Sichuan province of China and 10 patients who were diagnosed as metastasis disease during the same period for comparison purposes. Genomic DNA from lung cancers from individual patients was analyzed by six microsatellites (D2S1363, D6S1056, D7S1824, D10S1239, D15S822, and D22S689) with PCR to identify discordant allelic variation. The experiments were approved by the West China Hospital Ethics committee (No.2013 (33)) and all patients agreed to participate in the study and signed an informed consent form.

**Results:**

All of the 10 patients with distant metastasis showed a consistent consequence that we called “unique trend” between primary tumor and distant metastasis. The “trend” is representive in this study, which means that all alleles corresponding to six microsatellite markers were detected in DNA from primary tumors but were reduced or not observed in DNA from metastatic tumors. In the group of synchronous lung tumor with different histological types, the result showed a “contradictory trend”. Some alleles were detected in DNA from primary tumors but were reduced or not observed in DNA from metastatic tumors and other alleles corresponding to six microsatellite markers were detected in DNA from metastatic tumors but were reduced or not observed in DNA from primary tumors. In the third group (synchronous lung tumor with same histological types), 2 of 8 patients showed “unique trend” and the others showed “contradictory trend”.

**Conclusions:**

With polymorphic microsatellite markers, the “unique trend” that represents metastasis cancers and the “contradictory trend” that represents primary multiple tumors are useful in the diagnosis between tumors found at the same time in the pulmonary even diagnosed with the histopathological evaluation from a single patient.

## Background

Surgery-based multi-discipline treatment has significantly prolonged the survival for patients with lung cancer, and at the same time increased risk for multiple primary cancers after the first treatment. The incidence of multiple primary lung cancers has been reported to range from 0.7% to 15% of patients with lung cancer [1-5]. Dual primary lung cancer, also known as multiple primary lung cancer (MPLC), refers to two or more primary cancers in different sites of one or both lungs, with either consistent or different histology but no association between two cancers. Based on the time when the tumors are identified, the disease can be classified as synchronous or metachronous [6]. At present, diagnostic criteria are simply based on pathology and radiology, the majority of multiple primary lung cancers are misdiagnosed as metastatic cancer in the issue. They, however, present as a solitary mass in the majority of circumstances [7], raising the possibility of a metachronous second primary lung cancer. Especially, when patients develop multiple, morphologically similar lung cancer, the clinical diagnosis becomes critical for the selection of an appropriate treatment (surgery for multiple primary tumors versus systemic chemotherapy for solitary pulmonary metastatic disease). In the absence of carcinoma in situ, the morphologic similarity between the first and the second lung neoplasm can make it impossible to make the diagnostic distinction between metachronous primary neoplasms and solitary pulmonary metastasis, while it is also difficult to make the diagnostic distinction between synchronous primary lung cancer and the metastasis if they have the similar morphology. Thus, traditional histopathological assessment of neoplasms of the aerodigestive tract cannot definitively distinguish multiple primary cancers from metastatic disease when solitary, histologically similar cancers arise synchronously or metachronously in an individual patient. Comparison of the histology of both neoplasms for similarity or difference in histologic grade may suggest a common or independent origin, but it is recognized that tumor grade can show regional heterogeneity in an individual neoplasms, and that metastases may not always display the same grades as their parent primaries [8].

Synchronous lung cancers are rare and rather little is known as for their genetic basis [9]. The incidence of synchronous lung carcinomas is variably reported between 1% and 16% [10]. The 5-year survival of patients with synchronous multiple primary lung cancers has been reported to range from 0% to 44% despite an early diagnosis [11-14].

Recent advances in molecular biology have provided several markers that can be used for clonal analysis. As genomic instability represents one of the hallmarks of human cancer [15-17], the multiple, independently developing neoplasms in an individual patient will possess measurable genomic variation that can be analyzed to generate unique molecular signatures that reflect tumor lineage and relatedness. Allelic variation between neoplasms often reflects accumulation of differential chromosomal deletion events. These chromosomal deletions are tolerated (non-lethal), but distinct from the molecular alterations that drive tumorigenesis, which will be common to most/all tumors of a specific type. Comparison of molecular signatures between two (or more) tumors from a single individual facilitates identification of common and unique genetic alterations [8]. These genetic alterations can be detected by polymerase chain reaction (PCR)-based microsatellite analysis, by DNA sequencing, and occasionally by immunohistochemistry.

In this study, we examined 18 patients with synchronous multiple lung tumors utilizing a PCR-based approach to screen polymorphic microsatellite markers for variation in allele numbers. The results of this study demonstrate that molecular methods are useful for distinction of second primary neoplasms from solitary pulmonary metastasis in patients.

## Methods

### Patients and clinical features

Among the consecutive patients with primary lung cancer who had undergone a surgical resection between April 2003 and December 2012 at the Department of the Thoracic Surgery at West China Hospital, Sichuan, China, 13 patients were diagnosed with multiple primary lung cancers according to criteria proposed by Martini and Melamed [18]. Cases from patients with multiple lung cancers were retrieved and carefully reviewed. They were classified as synchronous (diagnosis of both tumors at the same time based solely on the traditional histopathological evaluation) tumors with the same histological type or not. Of these, 8 patients were diagnosed as having multiple primary lung cancers with the same histological types, and 5 patients were diagnosed with different histological types. 10 cases of pulmonary tumors with metastasis (to brain,Sternum or adrenal gland) were selected for comparison. All patients were followed up and the status recorded as alive or dead. In each case of the synchronous multiple primary lung cancers; the first tumor was designated as Tumor 1 (T1), while subsequent tumors were designated as Tumor 2 (T2). When multiple tumors were surgically removed from a given patient at the same time, tumor designations were assigned arbitrarily. While the multiple neoplasms from each patient were designated T1 and T2, the actual temporal order of appearance and lineage relationships between tumors (if any) were not known. Thus, in each patient these neoplasms could represent (i) multiple primary lung cancers, or (ii) primary lung cancer and derived metastatic lesions. The age at diagnosis, sex, site of tumor, histology, and the status of following up for each patient are given in Table 1. The experiments were approved by the West China Hospital Ethics committee (No.2013 (33)) and all patients agreed to participate in the study and signed an informed consent form.

**Table 1:** Clinical and pathologic data of all patients

	**Patients designation**		**Age**	**Sex**	**Site of tumor**	**Histology**	**Follow up**
**Metastatic carcinomas originating in the lung**	**Patient1**	T1	54	M	RLL	squamous carcinoma	Alive
		T2			Sternum	squamous carcinoma	
	**Patient2**	T1	59	M	Brain	adenocarcinoma	Dead
		T2			RUL	adenocarcinoma	
	**Patient3**	T1	70	M	LUL	squamous carcinoma	Dead
		T2			Brain	squamous carcinoma	
	**Patient4**	T1	45	F	LLL	adenocarcinoma	Dead
		T2			Brain	adenocarcinoma	
	**Patient5**	T1	39	F	RLL	adenocarcinoma	Alive
		T2			Brain	adenocarcinoma	
	**Patient6**	T1	58	F	RUL	adenocarcinoma	Alive
		T2			Rib	adenocarcinoma	
	**Patient7**	T1	56	M	Brain	adenocarcinoma	Dead
		T2			RML	adenocarcinoma	
	**Patient8**	T1	55	M	LUL	squamous carcinoma	Alive
		T2			Left adrenal gland	squamous carcinoma	
	**Patient9**	T1	46	M	LLL	adenocarcinoma	Alive
		T2			Brain	adenocarcinoma	
	**Patient10**	T1	56	M	LUL	squamous carcinoma	Alive
		T2			Brain	squamous carcinoma	
**Synchronous lung tumor with different histological types**	**Patient1**	T1	71	M	RUL	adenocarcinoma	Dead
		T2			RLL	squamous carcinoma	
	**Patient2**	T1	47	M	RLL	squamous carcinoma	Alive
		T2			RUL	adenocarcinoma	
	**Patient3**	T1	72	M	LLL	adenocarcinoma	Dead
		T2			LUL	squamous carcinoma	
	**Patient4**	T1	59	M	RUL	adenocarcinoma	Alive
		T2			RLL	squamous carcinoma	
	**Patient5**	T1	74	M	RUL	adenocarcinoma	Alive
		T2			RLL	squamous carcinoma	
**Synchronous lung tumor with same histological types**	**Patient1**	T1	53	M	RML	adenocarcinoma	Dead
		T2			RUL	adenocarcinoma	
	**Patient2**	T1	47	M	LLL	adenocarcinoma	Dead
		T2			LUL	adenocarcinoma	
	**Patient3**	T1	52	F	RUL	adenocarcinoma	Alive
		T2			RLL	adenocarcinoma	
	**Patient4**	T1	70	F	RLL	adenocarcinoma	Alive
		T2			RUL	adenocarcinoma	
	**Patient5**	T1	73	M	LLL	squamous carcinoma	Alive
		T2			LUL	squamous carcinoma	
	**Patient6**	T1	65	M	RML	squamous carcinoma	Alive
		T2			RLL	squamous carcinoma	
	**Patient7**	T1	55	M	LLL	squamous carcinoma	Alive
		T2			LUL	squamous carcinoma	
	**Patient8**	T1	69	M	RUL	adenocarcinoma	Dead
		T2			RLL	adenocarcinoma	

### Preparation of genomic DNA

Recut sections stained with hematoxylin and eosin (H&E) were used to identify regions of well-preserved tumor tissue containing ≥90% tumor. Tumor tissues from 3–5 unstained 5 μm sections were dissected away from tumor stroma and using a sterile scalpel for each case and collected in 600 μl of xylene. Deparaffinization was achieved by incubating for 5 min in xylene, followed by centrifugation at 14,000× g for 2 min to collect deparaffinzed tissues. The tissue pellet was resuspended in 600 μl of 95% ethanol, washed for 5 min, and centrifuged at 14,000× g for 3 min. The resulting tissue pellet was dried and DNA isolated using the Paraffin-embedded tissue genomic DNA extraction kit according to the manufacturer’s protocol (Bioteke Corporation, Beijing).

### Microsatellite PCR analysis

Genomic DNA collected from tumor samples were examined for 6 polymorphic microsatellite markers that are the most representative in the detection of measurable genomic variation and displayed high reproducibility in a PCR-based assay, including: D2S1363 (2q34), D6S1056 (6q23.2), D7S1824 (7q33), D10S1239 (10q24.3), D15S822 (15q12), and D22S689 (22q12.1). Information concerning these markers and primer sequences is available on the Genome Database (http://www.gdb.org/) and the NCBI genome database (http://www.ncbi.nlm.nih.gov/). The oligonucleotide primers corresponding to each microsatellite marker (MapPairs™primers) were purchased from Sangon Biotech (Shanghai) Co., Ltd. For PCR, the reaction samples were each prepared to a total volume of 25 μL as follows: 12.5 μL PCR Mix (2X) (PCR Master Mix 2X, Thermo SCIENTIFIC), 1 μL each primer, 1 μL template DNA, and 9.5 μL nuclease-free water. Amplifications were carried out in a Perkin Elmer Thermocycler using a step–cycle program consisting of 39 cycles of 95°C for denaturing (30 seconds), 55°C for annealing (30 seconds), and 72°C for extension (1 min). PCR products were fractionated on 6% polyacrylamide gels containing Tris–borate/EDTA (pH 8.0) and visualized by ethidium bromide staining. PCR reactions that did not produce detectable amplified products were re-amplified to confirm a negative result.

## Results

Molecular signatures were generated for each of 46 tumors using 6 polymorphic microsatellite markers. These 46 tumors represent either multiple primary cancers or primary cancer and metastatic disease from 13 patients (n = 26), as well as a known outgroup control primary lung cancer with its metastasis (n = 20). One objective of the current investigation was to identify microsatellite markers that are useful in the detection of measurable genomic variation (allelic imbalance or allelic variation) among multiple tumor-derived DNAs from individual patients. Of the 6 microsatellite markers evaluated, all detected allelic imbalance or allelic variation among all tumors from at least one patient. The 6 microsatellite markers examined identified allelic variation in 17–57% of patients, and the majority of microsatellite markers (13/23, 57%) detected allelic differences in tumors. These observed allelic differences correspond to evidence of dissimilarity among the tumors in question.

### Analysis of primary and metastatic tumors

**Figure 1:**
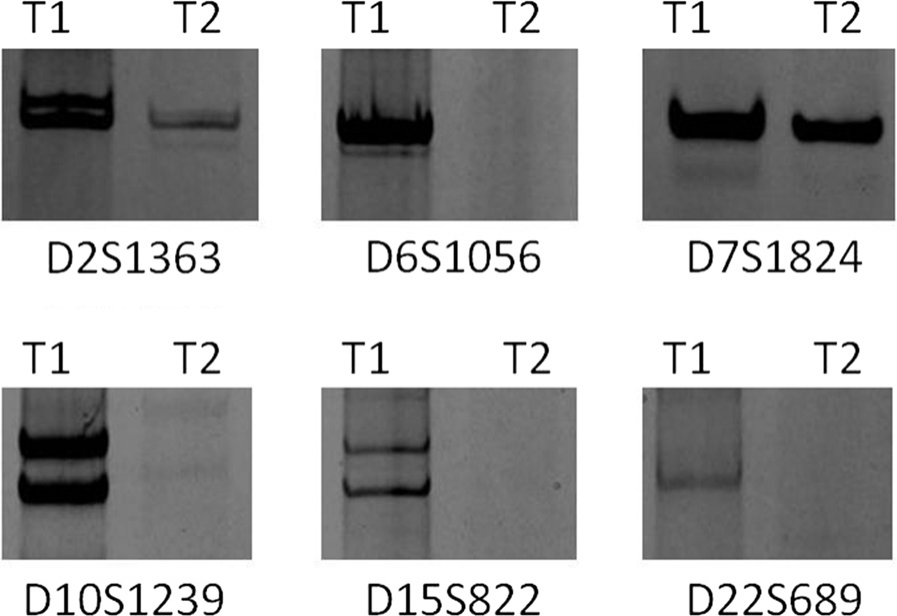
**Molecular analysis of Patient 6 from primary and metastatic tumors group.** Representative results of microsatellite PCR analysis of DNA from the neoplasms associated with Patient 6. T1 refers to the primary lung cancer and T2 refers to the metastatic tumor. The identity of each microsatellite marker analyzed is indicated.

To verify that microsatellite PCR could detect a lineage relationship between tumor pairs, we first analyzed the relationship between primary and metastatic tumors. Ten pairs of tumors were chosen, each consisting of a primary lung tumor and a metastatic tumor to the brain, the sternum or the left adrenal gland. (Table 2) Lineage relationships for each of these tumor pairs were determined by molecular analysis. For example, alleles corresponding to microsatellite markers D2S1363, D10S1239, and D15S822 were detected in DNA from T1 of Patient 1, but were not observed in DNA from T2 (Table 2). Likewise, alleles corresponding to microsatellite markers D10S1239 and D22S689 were observed in T1 of Patient 3, but were not detected in T2. Similar results were obtained for Patients 4, 8 and 10. In the case of Patient 5 and 9, the allelic variation was only observed at D22S689 or D15S822. Because the tumor designations were assigned arbitrarily when multiple tumors were surgically removed from a given patient at the same time, Patient 2 and 7 represented inverted consequence in the Table 2. However, we understand that the T2 of Patient 2 and 7 were primary tumors and T1 were metastatic tumors. In the case, Patient 6, the two neoplasms share common allelic patterns only for 1 microsatellite marker at D7S1824, but show differing allelic patterns for 5 microsatellite markers at D2S1363, D6S1056, D10S1239, D15S822 and D22S689 (see Figure 1 and Table 2). The molecular signatures for these two tumors are notably different and the pattern of allelic variation presented allelic loss in 5 microsatellite markers direction in T2 of the patient. All of these support the hypothesis that the lung cancer was the primary neoplasm that gave rise to the brain, sternum or left adrenal gland metastasis. Further, these molecular data indicate that the metastatic cancer in the brain, sternum or left adrenal gland could not have given rise to the lung cancer. These observations confirm that microsatellite PCR has utility for logically determining clonal relatedness and lineage among neoplasms in an individual patient.

**Table 2:** Microsatellite marker of all tumors

	**Patient designation**		**D2S1363**	**D6S1056**	**D7S1824**	**D10S1239**	**D15S822**	**D22S689**
**Primary and metastatic tumors**	**Patient1**	T1	**+**	**+**	**+**	**+**	**+**	**-**
		T2	**-**	**+**	**+**	**-**	**-**	**-**
	**Patient2**	T1	**-**	**-**	**-**	**-**	**++**	**-**
		T2	**-**	**+**	**+**	**+**	**++**	**-**
	**Patient3**	T1	**+**	**++**	**+**	**++**	**+**	**+**
		T2	**+**	**++**	**+**	**+**	**+**	**-**
	**Patient4**	T1	**+**	**+**	**+**	**++**	**+**	**-**
		T2	**+**	**+**	**+**	**-**	**-**	**-**
	**Patient5**	T1	**+**	**+**	**+**	**+**	**++**	**++**
		T2	**+**	**+**	**+**	**+**	**++**	**-**
	**Patient6**	T1	**++**	**+**	**+**	**++**	**++**	**+**
		T2	**+**	**-**	**+**	**-**	**-**	**-**
	**Patient7**	T1	**-**	**-**	**-**	**+**	**-**	**-**
		T2	**++**	**-**	**+**	**++**	**-**	**-**
	**Patient8**	T1	**+**	**++**	**+**	**+**	**+**	**-**
		T2	**+**	**-**	**+**	**+**	**-**	**-**
	**Patient9**	T1	**+**	**++**	**+**	**++**	**+**	**-**
		T2	**+**	**++**	**+**	**++**	**-**	**-**
	**Patient10**	T1	**++**	**++**	**+**	**+**	**-**	**-**
		T2	**-**	**-**	**+**	**+**	**-**	**-**
**Synchronous tumors with different histological types**	**Patient1**	T1	**+**	**+**	**+**	**-**	**-**	**+**
		T2	**+**	**+**	**+**	**++**	**-**	**-**
	**Patient2**	T1	**++**	**++**	**+**	**++**	**++**	**-**
		T2	**++**	**+++**	**+**	**+**	**++**	**-**
	**Patient3**	T1	**++**	**-**	**+**	**-**	**++**	**-**
		T2	**-**	**+**	**-**	**+**	**++**	**-**
	**Patient4**	T1	**-**	**-**	**+**	**+**	**-**	**-**
		T2	**++**	**-**	**+**	**-**	**-**	**-**
	**Patient5**	T1	**++**	**++**	**+**	**++**	**+**	**+**
		T2	**++**	**+**	**+**	**++**	**++**	**+**
**Synchronous tumors with same histological types**	**Patient1**	T1	**+**	**++**	**+**	**++**	**+**	**+**
		T2	**+**	**+**	**+**	**++**	**-**	**-**
	**Patient2**	T1	**++**	**+**	**+**	**+**	**++**	**+**
		T2	**+**	**-**	**+**	**+**	**-**	**-**
	**Patient3**	T1	**++**	**++**	**+**	**++**	**++**	**+**
		T2	**++**	**++**	**+**	**++**	**++**	**-**
	**Patient4**	T1	**++**	**-**	**+**	**-**	**-**	**-**
		T2	**-**	**-**	**+**	**++**	**-**	**-**
	**Patient5**	T1	**+**	**+**	**+**	**+**	**-**	**-**
		T2	**+**	**+**	**-**	**+**	**++**	**+**
	**Patient6**	T1	**+**	**+++**	**+**	**+**	**-**	**-**
		T2	**+**	**+**	**+**	**-**	**-**	**-**
	**Patient7**	T1	**-**	**++**	**+**	**+**	**-**	**-**
		T2	**-**	**-**	**+**	**+**	**-**	**-**
	**Patient8**	T1	**+**	**+**	**+**	**+**	**-**	**-**
		T2	**+**	**-**	**+**	**-**	**-**	**-**

T1 refers to the first neoplasm; T2 refers to the second neoplasm; + indicates number of alleles amplified; - indicates a lack of allele amplification.

### Analysis of synchronous lung tumor with different histological types

**Figure 2:**
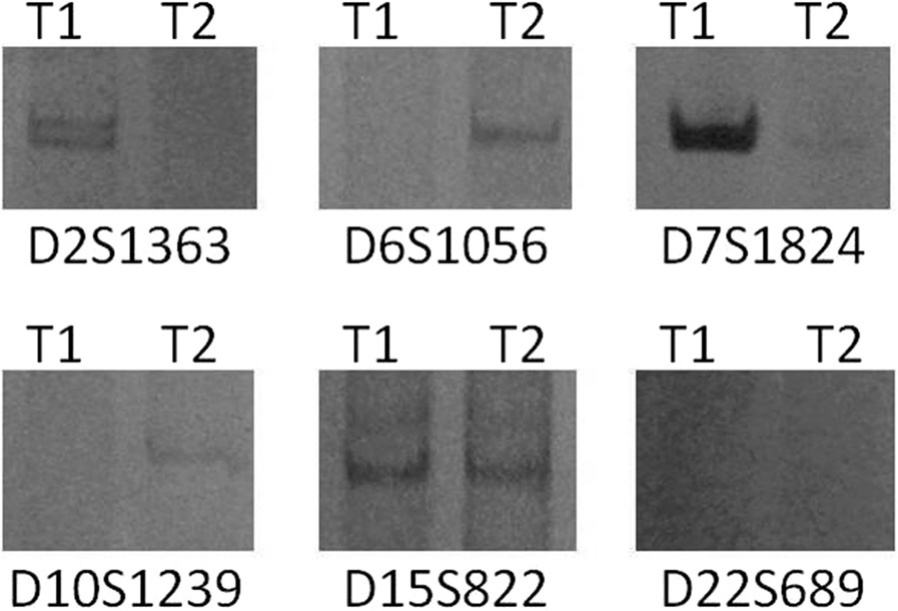
**Molecular analysis of Patient 3 from synchronous lung tumor with different histological types group.** T1 refers to the lung cancer of adenocarcinoma and T2 refers to the lung cancer of squamous carcinoma. The identity of each microsatellite marker analyzed is indicated.

Five cases of synchronous tumors (Table 2) of the lung were also studied for molecular analysis. In the typical case of Patient 3, the observed allelic variation at D2S1363 and D7S1824 suggest that T2 could be derived from T1, consistent with metastatic disease (see Figure 2 and Table 2). However, the allelic variation noted at D6S1056 and D10S1239 contradicts this possible lineage relationship. Thus, the collective allelic variation involving 4 microsatellite markers suggests that neoplasms from Patient 3 represent independently arising primary lung cancer. In the other patients, alleles corresponding to microsatellite markers D10S1239 were amplified in DNA from T2 of Patient 1, but were not observed in DNA from T1. However, in the same patient, D22S689 were detected in DNA from T1 of Patient 1, but were not found in DNA from T2. Similar results were obtained for Patients 2, 4 and 5. However, this conclusion is made with less confidence when the numbers of discordant changes are this few. Given that there is concern about the effects of intra-tumor heterogeneity on this type of analysis, increased numbers of microsatellite markers (and observations of allelic variation) might be required to draw clear conclusions in some cases.

### Analysis of synchronous lung tumor with same histological types

Eight cases presented with synchronous multiple tumors of the lung were also studied for molecular analysis. The microsatellite PCR results for each of these patients are given in Table 2.

Patient 5 was diagnosed with adenocarcinoma of the right upper lobe and right lower lobe at the same time. Given the histopathology and interval for these neoplasms, conventional staging criteria would classify one of the lung cancer as a metastatic lesion derived from the other one. However, molecular analysis of these tumors identified discordant allelic variations involving 3 different microsatellite markers, 2 of which are gains of alleles, arguing that these cancers arose independently. Alleles for D7S1824 was detected in the T1 that were not observed in the subsequent lung tumor (T2). In contrast, alleles corresponding to D15S822 and D22S689 were detected in the lung cancer (T2) that were not observed in the T1. These mutually exclusive allelic losses strongly suggest that these neoplasms are not related. Thus, the lung cancer in this patient represents a second primary carcinoma rather than a solitary metastatic lesion derived from the other one. Likewise, alleles corresponding to microsatellite markers D2S1363 was observed in T1 of Patient 4, but was not detected in T2. At the same time, microsatellite markers D10S1239 was observed in T2, but was not detected in T1. The results of two patients are similar to the synchronous lung tumor with different histological types and suggest that the synchronous lung tumors in this patient are not related and represent multiple primary tumors.

Patient 1 was diagnosed as adenocarcinoma of the right middle lobe and right upper lobe. In this case, the molecular evidence was based upon concordant results with D6S1056, D15S822 and D22S689. Alleles corresponding to microsatellite markers D6S1056, D15S822 and D22S689 were observed in T1, but were not detected in T2. The results strongly suggest that the T1 has given rise to T2 and suggest that the T1 metastasized to the T2, based upon gain of alleles for these markers.

Molecular analysis of Patient 2 provided a clear example of a patient with clonally-related neoplasms even it was classified as synchronous lung tumor, where the evidence suggest that the T1 metastasized to the T2. In this case, microsatellite markers D2S1363, D6S1056, D15S822 and D22S689 were detected in DNA from T1, but these markers were not found in DNA from T2.

Like Patient 4 and 5, either primary tumor in Patient 6 and 8 could have given rise to the other lung cancer (in the case of metastatic disease), or it could represent a primary synchronous cancer. However, the results with D6S1056 and D10S1239 suggest that T1 have given rise to T2, based upon gain of alleles for these markers in the T1. These observations argue that the lung cancer in patient 6 and 8 represent a metastatic disease rather than primary synchronous lung cancer.

Patient 3 and 7 was diagnosed as the synchronous lung tumor with same histological types according to the conventional clinical staging criteria. The goal was to determine if T1 did not relate to T2. Alleles for D6S1056 or D22S689 were detected in the DNA from the T1 that was not observed in the T2 (Table 2), consistent with the suggestion that the T2 represents a metastatic lesion derived from the T1. Thus, the molecular evidence suggests that the Patient 3 and 7 likely represent metastasis cancers, rather than solitary primary tumors. However, this conclusion is made with less confidence when the numbers of discordant changes are this few.

## Discussion

When patients present with multiple, morphologically similar lung cancer, it can be impossible to distinguish whether the neoplasms are clonally related (reflecting metastatic disease) or clonally unrelated (representing multiple primary cancer).

According to current histopathologic criteria, only dual tumors with different histology or in different organs will be diagnosed as multiple primary lung cancer. The problems with this in clinical setting includes: (1) all the tumors with consistent histopathology are diagnosed as metastatic cancer, but tumors with different genotypes should be actually diagnosed as multiple cancer; (2) synchronous lung tumors in one or different lobes are simply defined as satellite lesion (T4) or metastatic lesion (M1); (3)when the interval between the first and the second tumor is ≤ 2 years, metachronous multiple primary lung cancer with consistent histology is recommended to be diagnosed as metastatic cancer;when the interval is > 4 years, it is to be diagnosed as multiple cancer; and when the interval is > 2 years and ≤ 4 years, its diagnosis is still inconclusive; (4) even when histology of two tumors is different, the possibility of metastatic or recurrent tumors cannot be excluded given consideration to the heterogeneity of tumors; and (5) the differential diagnosis of intrapulmonary metastatic lung cancer (via bloodstream dissemination) verse primary lung cancer is also perplexing in clinical setting; however, tumors with consistent histology, and N2 and N3 lymph nodes or multiple-organ metastasis and those with consistent histology and < 2 years interval are often diagnosed as intrapulmonary metastasis [6]. A critical problem is that surgical treatment is a first-choice and effective strategy for multiple primary lung cancer, with a treatment efficacy comparable to that in first primary cancer; but in current clinical setting, the second tumor will usually be diagnosed and treated as metastatic cancer based on radiology, as a result, patients with multiple primary lung cancer lose the opportunity for surgical and other proper treatments. At the same time, it is difficult to obtain the specimens (or the specimens were not harvested at all) for histological classification. Such misdiagnosis poses huge damage on the patients, both physically and mentally, and probably in a fatal way. Therefore, one way to tackle this predicament is to explore new practical techniques and markers to diagnose and prognosis for multiple primary lung cancers.

The exact pathogenic mechanism of multiple primary lung cancer is still unclear, but for the time being, field cancerization is one important hypothesis to explain the occurrence of multiple primary malignant tumors. According to the hypothesis, the organ systems exposed to the same carcinogen have increased chance for developing malignant tumors [19]. Examples are synchronous lung cancers, head-and-neck cancer or bladder cancer, because smoking as a carcinogen has important role in the etiology of these malignant tumors [20]. Haraguchi et al. [21] demonstrated in their study that multiple primary lung cancers tended to be inherited in family; lung cancer patients with family history had higher chance for a second or third primary tumor than those without family history. Latest studies found that genomic instability and changes in gene expression profile (such as tumor suppressor genes and DNA repairing genes [19]) and even mutation and deletion of chromosomes were closely related to the occurrence of multiple primary cancers. Analysis of primary lung tumors and their corresponding metastases revealed identical point mutations affecting p53 in the tumor/metastases pairs in 73% of the cases, suggesting that mutation of p53 is an early event that is essential for tumorigenesis and conserved during progression to metastatic disease [22-24]. These studies from the literature provide strong evidence based upon p53 mutation status that many second neoplasms of the aerodigestive tract arise independently and therefore represent multiple primary tumors affecting an individual patient. However, the methodology employed (based upon characterization of p53 mutation status) is not practical for broad application in the molecular diagnostic evaluation of patients with multiple neoplasms. Practical molecular testing of patients affected by multiple neoplasms requires a method that is less time-consuming and less expensive. Other methods have been applied to this problem, including determination of tumor relatedness through analysis of allelic loss of heterozygosity (LOH). Froio et al. [25] labeled and analyzed the LOHs of the 40 chromosomes in a patient with three synchronous multiple primary cancers and found different genetic labels for three tumors, suggesting that three tumors were independent on each other and that genetic markers were useful in diagnosing multiple cancers. While this approach is effective in the identification of molecular differences between tumors, it requires the presence of normal tissue for complete analysis [26]. This is problematic since germline DNA or normal (non-neoplastic) tissue may not always be available for analysis, especially when a tumor biopsy is collected and analyzed. For this reason, development of a molecular diagnostic test that relies solely on the collection of tumor tissue from either a biopsy or needle aspirate will prove to be more practical in a clinical setting.

Mercer et al. [8] conducted a study on lung metastatic carcinoma from head-and-neck squamous cell carcinoma and primary lung carcinoma. They refined a molecular method to analyze multiple tumors that does not rely on collection of normal tissue, can be performed with minimal tumor sample, and will complement clinical criteria for diagnostic discrimination between multiple primary cancer versus solitary metastatic disease. At last, they found that detections of microsatellite alterations and deletion sites in tumor cell DNA could be used as diagnostic and prognostic markers for multiple cancers.

In our study, we wanted to discriminate and analyse the characteristic of multiple synchronous lung cancers and metastatic lung cancers that without relying on collection of normal tissure. We collected 46 specimens of primary pulmonary synchronous tumors and metastatic tumors and compared the genetic profiles of them.

Results from the metastatic tumors serve to validate that clonal tumor cells have similar profiles. As the consequences showed of all the patients in our study, especially in patient 1, 6 and 7, alleles corresponding to microsatellite markers D2S1363, D6S1056, D7S1824, D10S1239, D15S822, and D22S689 were detected in DNA from primary tumors, but were reduced or not observed in DNA from metastatic tumors. The characteristic of this “unique trend” is representative in our study.

The next groups of tumors we studied were synchronous tumors with different histological types of the lung. Among the five synchronous pulmonary tumors, they represent true synchronous tumors according to the clinical decision rule of Martini N and Melamed MR [18]. The result showed a “contradictory trend” comparing with the first study group. The paired tumors in cases 3 appeared to be typical of the five patients. The observed allelic variation at D2S1363 and D7S1824 suggest that T2 could be derived from T1, consistent with metastatic disease. However, the allelic variation of D6S1056 and D10S1239 were noted in T2 but not observed in T1, which means that T1 could be also derived from T2, so that the result contradicts the possible lineage relationship of metastatic disease.

Among the eight synchronous tumors with same histological types of the lung, the results of cases 4 and 5 present typical “contradictory trend” and were similar to the synchronous lung tumor with different histological types. It suggests that the synchronous lung tumors in this patient are not related and represent multiple primary tumors. However, the controversial conquences happened in patient 1, 2, 3, 6, 7 and 8. According to the Martini N and Melamed MR [18], all of these six patients were synchronous lung cancers. Withing the result of this study, however, the analysis of them showed reverse. As the result showed before, Patient 2 provided a clear example of a patient with clonally-related tumors, where the evidence suggest that the T1 metastasized to the T2. In this case, microsatellite markers D2S1363, D6S1056, D15S822 and D22S689 were detected in DNA from T1, but these markers were not found in DNA from T2. The “trend” was similar to the group of primary and metastatic tumors which was considered as intrapulmonary metastasis. The observations of patient 6 and 8 were also representing intrapulmonary metastasis, based upon the “trend”. The molecular evidence suggests that the Patient 3 and 7 in this group likely represent metastasis cancers, rather than solitary primary tumors. However, this conclusion is made with less confidence when the numbers of discordant changes are this few.

## Conclusion

The results presented in this study demonstrate that molecular analysis of allelic variations at polymorphic microsatellite markers can be used to determine lineage relationships between multiple tumors, facilitating the discrimination of second primary cancer versus metastatic disease. This approach is rapid and sensitive. For each of the patients diagnosed as multiple lung cancers, especially in the patients with the same histological type, it was impossible to determine an appropriate diagnosis of multiple primary cancer versus metastatic disease based solely on the traditional histopathological evaluation of their tumors. Performance of a comparative PCR-based molecular diagnostic test requires that DNA samples corresponding to the first cancer be retrieved for analysis along with the subsequently arising tumor (or tumors). Thus, it is paramount that such a test be amenable to the utilization of DNA samples from formalin-fixed paraffin-embedded tissues since these may be the only available source of DNA for the prior cancer. At last, with polymorphic microsatellite markers, the “unique trend” that represents metastasis cancers and the “contradictory trend” that represents primary multiple tumors are useful in the diagnosis between tumors found at the same time in the pulmonary even diagnosed with the histopathological evaluation. Precise determination of the clonal origin of multiple lung tumors might help rationalize treatment strategy and hopefully might improve prognosis of the affected patients.

## Competing interests

The authors declare that they have no competing interests.

## Authors’ contributions

CS was involved in drafting the manuscript. HX, LL, YZ and DC made contributions to the concepts, acquisition and analysis of the data. HD and ZH were involved in acquisition of data and preparing the figures. GC designed and revised the manuscript. All authors have read and approved the final manuscript.

## Pre-publication history

The pre-publication history for this paper can be accessed here:

http://www.biomedcentral.com/1471-2407/13/467/prepub
